# Analysis of renal lesions in Chinese tuberous sclerosis complex patients with different types of *TSC* gene mutations

**DOI:** 10.1590/1678-4685-GMB-2020-0387

**Published:** 2022-05-27

**Authors:** Wenda Wang, Yang Zhao, Xu Wang, Zhan Wang, Yi Cai, Hanzhong Li, Yushi Zhang

**Affiliations:** 1 Chinese Academy of Medical Sciences and Peking Union Medical College, Peking Union Medical College Hospital, Department of Urology, Beijing, China.; 2 Central South University, Xiangya Hospital, Department of Urology, Changsha City, China.

**Keywords:** Tuberous sclerosis complex (TSC), renal lesions, *TSC1* mutations, *TSC2* mutations

## Abstract

We sought to explore the relationship between renal lesion features and genetic mutations in tuberous sclerosis complex (TSC) patients. TSC patients with renal lesions were subjected to *TSC1/2* gene next-generation sequencing (NGS). *TSC1/2* mutation types and imaging examinations were screened for combined analysis of genetic and clinical features. Seventy-three probands among TSC patients with renal lesions were included. Twenty affected relatives were also included. In total, 93 patients were included. Eighty patients (86.0%) had bilateral renal angiomyolipomas (AMLs), and one had epithelioid AML. Two patients had polycystic kidney disease, one had renal cell carcinoma, and one had Wilms tumor. Among the 73 probands, four had *TSC1* mutations, 53 had *TSC2* mutations, and 16 had no mutations identified (NMI). There was no statistically significant difference between *TSC1* mutation, *TSC2* mutation and NMI group (*P*= 0.309), or between familial and sporadic groups (*P*= 0.775) when considering AML size. There was no statistically significant difference between pathogenic/likely pathogenic and benign/likely benign/NMI groups (*P*= 0.363) or among patients with different mutation types of *TSC2* (*P*= 0.906). The relationship between the conditions of TSC gene mutations and the severity of renal lesions still needs more analysis. Patients with NMI, particularly those with familial disease, need more attention because the pathogenesis remains unknown.

## Introduction

Tuberous sclerosis complex (TSC) is an autosomal dominant genetic disorder characterized by hamartomas in organs including the brain, kidney, lung, skin, and heart (Sasongko et al., 2016). The birth incidence of TSC has been estimated to be approximately 1 in 6000 (Osborne et al., 1991). Renal lesions are the most common cause of death in adult TSC patients. These renal diseases of TSC may occur in early childhood and progress into adulthood (Lam et al., 2018). The most common kidney manifestation of TSC is angiomyolipoma (AML), which occurs in 70-90% of TSC patients (Northrup *et al.*, 2013). The other kinds of lesions include renal cysts and renal cell carcinomas (RCCs).

Approximately 75-90% of patients who meet TSC standard clinical criteria harbor *TSC1* or *TSC2* mutations (Tyburczy et al., 2015), and approximately 60-70% of TSC cases are sporadic (Sampson et al., 1989; van Slegtenhorst *et al.*, 1997). However, 10-15% of patients show no *TSC1* or *TSC2* mutations (also known as no mutation identified, NMI), despite with a clinical diagnosis. Researchers have reported that patients with *TSC2* mutations exhibit more severe clinical features than patients with other genetic changes (Dabora et al., 2001; Sancak et al., 2005; Camposano et al., 2009; Boronat et al., 2014), though there are relatively few studies focusing on the relationship between TSC gene mutations and TSC renal lesions. Here, we report information on genetic mutations in TSC patients with renal lesions and discuss the relationship between renal lesions and TSC mutations, including mutated genes and mutation types.

## Subjects and Methods

### Participants

We retrospectively searched TSC patients with renal lesions among outpatients who came to the Urology Department of Peking Union Medical College Hospital (PUMCH) from January 1st, 2015, to July 1st, 2020. The diagnosis of TSC was made based on the clinical diagnostic criteria of the 2012 international tuberous sclerosis complex consensus conference (Northrup *et al.*, 2013) or *TSC1/2* genetic diagnosis. TSC patients with renal lesions who received next-generation sequencing (NGS) of *TSC1/2* genes (including those who performed in the Outpatient Department or previously) and imaging examinations were screened for analysis of genetic and clinical features. When a patient was diagnosed with TSC, if more than one family member was clinically diagnosed with TSC and had the same TSC-associated pathogenic variant, familial TSC was recorded. When other familial members of NMI patients had the same NGS results and met TSC clinical diagnostic criteria, familial TSC was also confirmed. All familial members were included for further analysis. There was overlap between the samples in the present study and in the study of Cai et al. in 2017 (Cai *et al*., 2017). We recorded the maximal diameter at the largest cross-section of the largest lesion in each patient upon diagnosis. Our study was approved by the Ethics Committee of Peking Union Medical College Hospital. Written informed consent was obtained from all subjects for genetic tests and clinical information analysis. All methods were performed in accordance with the principles of the Declaration of Helsinki and all local regulations.

### NGS and mutation analysis

Genomic DNA was extracted from peripheral blood leukocytes using a QIAamp DNA Blood Mini Kit (Qiagen, Hilden, Germany) and fragmented into 200~250-bp fragments and purified using an Agencourt AMPure XP kit (BGI-Shenzhen, Shenzhen, China). After modification, ligation-mediated polymerase chain reaction (PCR) and purification were conducted, followed by the hybridization reaction using customized gene fragment-capturing chips (Roche NimbleGen, Madison, WI). Amplification with high-fidelity DNA polymerase and high-throughput sequencing of qualified DNA samples were carried out for continuous bidirectional sequencing of 90 cycles. Illumina base calling software (V. 1.7, Illumina) was used to analyze the original imaging data, and Burrows-Wheeler Aligner software (BGI-Shenzhen, Shenzhen, China) was employed for sequence alignments of qualified raw reads, which had been conducted using sequencing quality assessment. The bam data were used to assess read coverage in the target region and sequencing depth computation, single nucleotide variant (SNV) and insertion-deletion calling, and copy number variation detection. NGS of *TSC1* and *TSC2* was performed for gene coding regions with adjacent ±10-bp intron sequences. The sequences of the *Homo sapiens* hamartin and tuberin proteins were obtained from the National Center for Biotechnology Information database. Mutations in the *TSC1* or *TSC2* gene were compared with those in Tuberous Sclerosis Database. The reference sequences of *TSC1* (Chr9:132,891,348-132,945,268) and *TSC2* (Chr16:2,047,803-2,089,490) are NM_000368 and NM_000548, respectively. First, SNVs and insertion-deletions were called using SOAPsnp software (BGI-Shenzhen, Shenzhen, China) and Samtools pileup software (BGI-Shenzhen, Shenzhen, China), respectively. After probable causative mutations were found, Sanger sequencing to verify the mutations was performed for the participants and their affected family members. Second, if a single nucleotide polymorphism (SNP) frequency was more than 0.05 in any of 4 databases (dbSNP, HapMap, 1000 Genomes Project, and BGI local database), it was regarded as a polymorphism and not a causative mutation. Large rearrangements could be detected by NGS based on the read depth (RD) algorithm. When decreased sequencing depth in a region was detected, a large rearrangement was suspected. Then, PCR was used to confirm large rearrangements. Pathogenic variants were assessed under the protocol issued by ACMG using InterVar (Li and Wang, 2017) and ClinVar. All mutations were retrieved from Leiden Open Variation Database (LOVD), OMIM and ClinVar for labeling as already reported or novel. The possible impact of the identified mutations on protein function as a result of an amino acid substitution was examined using the online tools SIFT and PolyPhen-2.

### Statistical analysis

All statistical analyses were performed using SPSS 19.0 software (SPSS Inc., USA). Data are expressed as means ± standard deviation (mean ± SD) or n (%), as appropriate. Student’s unpaired *t* test or Tukey’s test was used to determine the differentiation state of continuous variables between different groups. Chi-Square or Fisher’s exact tests was used for comparison of dichotomic variables between different groups. A *P* value of less than 0.05 was considered statistically significant.

## Results

**Figure 1-:**
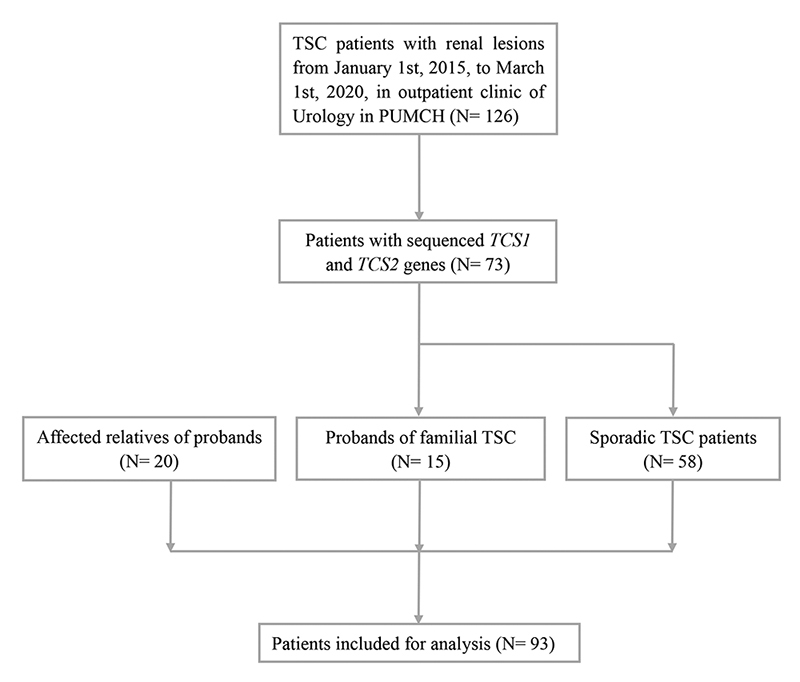
The flow chart for patients’ inclusion.

**Figure 2 -:**
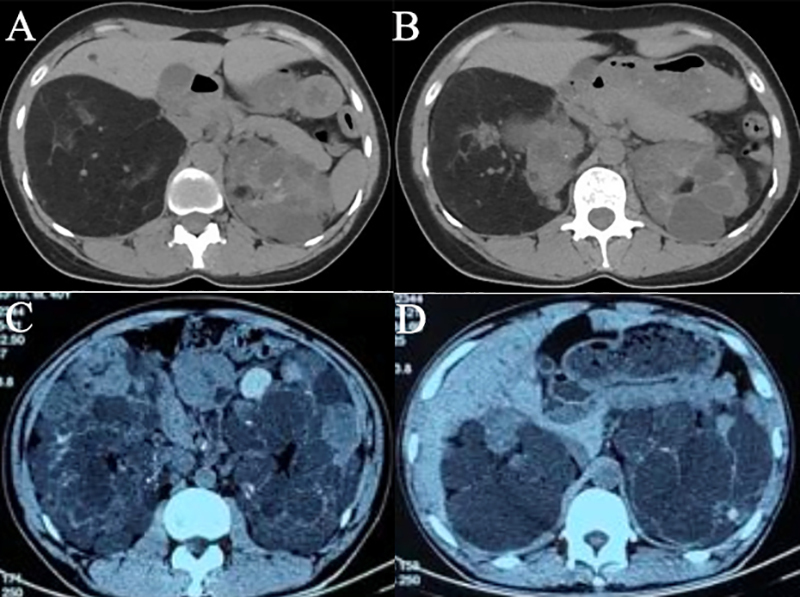
Computed tomography (CT) exam results: A and B, in a 35-year-old female patient with TSC2 EX2_42 DEL (had both *TSC2* and *PKD1* mutations), left kidney with multilocular cysts typical of polycystic kidney disease (PKD), and right kidney presenting a huge angiomyolipoma (AML) with a maximal diameter of 106mm; C and D, in a 40-year-old male patient with the same genetic variant, the presence of PKD bilaterally, without any specific signs of AML.

In total, 126 TSC patients with renal lesions were retrospectively analyzed from January 1st, 2015, to March 1st, 2020, in PUMCH. Among them, 73 patients underwent NGS (Table 1). Fifteen patients (20.5%) were probands of TSC families (2 *TSC1*, 11 *TSC2*, and 2 NMI). When all the members of familial TSC patients were included, there were 93 patients in total (Figure 1). The average age of the 93 patients was 28.4±10.0 years old. There were more female patients, with a male-female ratio of 1:1.5. Among all the 93 patients analyzed, 80 (86.0%) had bilateral renal AMLs, and one had a pathological diagnosis of epithelioid AML. The epithelioid AML patient received surgical resection due to rapid progression. One patient among these AML patients also exhibited the phenotype of polycystic kidney disease (PKD) (Figure 2A and B), and the patient had both *TSC2* and *PKD1* mutations. However, there was also another patient with the same mutations presented PKD only (Figure 2 C and D). Other renal lesions included RCCs in one patient and Wilms tumors in one patient, respectively (Table 2).

**Table 1 -:** *TSC1* and *TSC2* mutations in the 73 probands.

*TSC1*	4/73 (5.5%)
Nucleotide mutation	
Nonsense	3/4 (75.0%)
Fragment deletion	1/4 (25.0%)
*TSC2*	53/73 (72.6%)
Nucleotide mutation	
Nonsense	19/53 (35.9%)
Missense	7/53 (13.2%)
Frameshift	15/53 (28.3%)
Splicing	4/53 (7.5%)
Silent	1/53 (1.9%)
Fragment deletion	7/53 (13.2%)
NMI	16/73 (21.9%)

Notes: TSC, tuberous sclerosis complex; NMI, no mutation identified.

**Table 2 -:** Clinical characteristics of the 93 patients.

	All (n= 93)	TSC1 (n= 6)	TSC2 (n= 68)	NMI (n= 19)	*P*
Age	28.4±10.0 (5~57)	29.5±15.6 (6~44)	28.1±10.1 (8~57)	29.1±7.4 (5~38)	0.902
Sex					
Male	37	5	27	5	0.045
Female	56	1	41	14	
Familial TSC	15 (35)	2 (4)	11 (26)	2 (5)	0.202
Renal lesions					
AML	80	2	61	17	0.042
Epithelioid AML	1	0	1	0	-
AML with polycystic kidney disease	1	0	1	0	-
PKD (without AML)	1	0	1	0	-
Renal cell carcinomas	1	1	0	0	-
Wilms tumor	1	1	0	0	-
Renal AML diameter					
Diameter_max_ (mean±SD, mm)	101.8±59.1	58.5±29.0	107.3±60.6	86.9±53.5	0.309
Diameter_max_≥ 4cm	58	1	47	10	0.176

Notes: TSC, tuberous sclerosis complex; AML, angiomyolipoma; PKD, polycystic kidney disease. * Two patients had both *TSC2* and *PKD1* mutations, with renal lesions of PKD only and AML with PKD respectively.

Among the 73 probands of TSC patients, four carried *TSC1* gene mutations (Table 3). The patient with RCCs harbored a nonsense mutation (c.2227C>T) in the *TSC1* gene. The patient with Wilms tumors had a fragment deletion of *TSC1*. Fifty-three patients showed *TSC2* gene mutations (Table 4). Among them, seven were missense mutations. one was in the N-terminal TSC1-interacting region (residues 55 to 469), three were in the tuberin type domain (residues 555 to 903), and two were in the GTPase activator domain (residues 1562 to 1748). No *TSC1* or *TSC2* gene mutations were detected in 16 patients with a clinical diagnosis. 

**Table 3 -:** *TSC1* gene mutation data.

Site	Mutation type	Protein change	Lesions	Familial or not	Pathogenicity	Status	AML maximal diameter of proband (mm)
Nucleotide mutation							
c.733C>T (*)	Nonsense	p.Arg245Ter	AML	Yes (2)	Pathogenic	Reported	79.0
c.1372C>T (*)	Nonsense	p.Arg458Ter	AML	Yes (2)	Pathogenic	Reported	38.0
c.2227C>T (*)	Nonsense	p.Gln743Ter	RCC	No	Pathogenic	Reported	-
Fragment deletion							
EX9_12DEL (*)	-	-	Nephroblastoma	No	Likely pathogenic	Novel	-

Notes: AML, angiomyolipoma; RCC, renal cell carcinoma. *The overlapped cases between the present study and the study of Cai et al. (2017). The number of affected family members was labeled.

**Table 4 -:** *TSC2* gene mutation data.

Site	Mutation type	Protein change	Lesions	Familial or not	Pathogenicity	Status	AML maximal diameter of proband (mm)
Nucleotide mutation							
c.658C>T	Nonsense	p.Gln220Ter	AML	No	Pathogenic	Reported	164.5
c.1108C>T	Nonsense	p.Gln370Ter	AML	No	Pathogenic	Reported	147.0
c.1507C>T (*)	Nonsense	p.Gln503Ter	AML	No	Pathogenic	Reported	54.9
c.1513C>T	Nonsense	p.Arg505Ter	AML	No	Pathogenic	Reported	83.6
c.1513C>T	Nonsense	p.Arg505Ter	AML	No	Pathogenic	Reported	31.0
c.1874C>G	Nonsense	p.Ser625Ter	AML	No	Pathogenic	Reported	60.9
c.2194C>T	Nonsense	p.Gln732Ter	AML	No	Pathogenic	Reported	88.0
c.2194C>T	Nonsense	p.Gln732Ter	AML	No	Pathogenic	Reported	38.0
c.2590C>T	Nonsense	p.Gln864Ter	AML	No	Pathogenic	Reported	67.3
c.3412C>T	Nonsense	p.Arg1138Ter	AML	No	Pathogenic	Reported	204.0
c.3412C>T	Nonsense	p.Arg1138Ter	AML	No	Pathogenic	Reported	106.5
c.3412C>T (*)	Nonsense	p.Arg1138Ter	AML	No	Pathogenic	Reported	103.3
c.3412C>T	Nonsense	p.Arg1138Ter	AML	No	Pathogenic	Reported	-
c.3581G>A	Nonsense	p.Trp1194Ter	AML	No	Pathogenic	Reported	142.0
c.3685C>T	Nonsense	p.Gln1229Ter	AML	No	Pathogenic	Reported	105.0
c.3685C>T	Nonsense	p.Gln1229Ter	AML	Yes (2)	Pathogenic	Reported	46.9
c.3750C>G (*)	Nonsense	p.Tyr1250Ter	AML	No	Pathogenic	Reported	193.0
c.4129C>T (*)	Nonsense	p.Gln1377Ter	AML	Yes (2)	Pathogenic	Reported	107.8
c.4255C>T (*)	Nonsense	p.Gln1419Ter	AML	No	Pathogenic	Reported	87.6
c.856A>G	Missense	p.Met286Val	AML	No	Benign	Reported	-
c.1831C>T	Missense	p.Arg611Trp	AML	No	Pathogenic	Reported	30.2
c.1831C>T	Missense	p.Arg611Trp	AML	No	Pathogenic	Reported	96.0
c.2032G>A	Missense	p.Ala678Thr	AML	No	Benign	Reported	-
c.3475C>T	Missense	p.Arg1159Trp	AML	Yes (2)	Benign	Reported	-
c.5024C>T (*)	Missense	p.Pro1675Leu	AML	No	Pathogenic	Reported	202.1
c.5126C>T (*)	Missense	p.Pro1709Leu	AML	No	Pathogenic	Reported	116.5
c.2367C>T (*)	Silent	p.Val789Val	AML	No	Likely benign	Reported	49.0
c.203_204insA (*)	Frameshift	p.Ala68AlafsX7	AML	Yes (4)	Likely pathogenic	Novel	108.3
c.788_789insC (*)	Frameshift	p.Leu263LeufsX75	AML	Yes (2)	Likely pathogenic	Novel	106.4
c.788_789insC	Frameshift	p.Leu263LeufsX75	AML	No	Likely pathogenic	Novel	-
c.1201_1202insA	Frameshift	p.His401GlnfsX9	AML	Yes (3)	Likely pathogenic	Novel	266.0
c.1047dup	Frameshift	p.Arg350Ter	AML	Yes (2)	Pathogenic	Reported	33.4
c.1762_1763delGAinsT	Frameshift	p.Glu588Terfs	AML	No	Likely pathogenic	Novel	-
c.1852del	Frameshift	p.Leu618CysfsX80	AML	No	Likely pathogenic	Reported	204.0
c.2319delA (*)	Frameshift	p.Leu773LeufsX56	AML	No	Likely pathogenic	Novel	218.4
c.2233_2234del	Frameshift	p.Lys745AspfsX16	AML	Yes (2)	Likely pathogenic	Reported	59.0
c.2738_2739insT (*)	Frameshift	p.Thr913ThrfsX2	AML	No	Likely pathogenic	Novel	130.9
c.3601_3602insGGCCC (*)	Frameshift	p.Thr1203GlyfsX9	AML	No	Likely pathogenic	Novel	171.3
c.3683_3684insG (*)	Frameshift	p.Leu1228LeufsX6	AML	No	Likely pathogenic	Novel	60.0
c.4006_4007insC (*)	Frameshift	p.Ser1336SerfsX78	AML	No	Likely pathogenic	Novel	64.4
c.4544_4547del	Frameshift	p.Asn1515SerfsX60	AML	Yes (2)	Pathogenic	Reported	113.0
c.4926delC (*)	Frameshift	p.Asn1643ThrfsX29	AML	No	Likely pathogenic	Reported	202.0
c.976-1G>A	Splicing	-	AML	No	Pathogenic	Reported	58.0
c.1444-1G>C	Splicing	-	AML	No	Likely pathogenic	Reported	100.0
c.1947-1G>C (*)	Splicing	-	AML	No	Likely pathogenic	Reported	146.4
c.2098-2A>G (*)	Splicing	-	AML	No	Likely pathogenic	Reported	-
Fragment deletion							
EX2_16 DEL (*)	-	-	AML	No	Likely pathogenic	Novel	76.4
chr16:2098173-2138668 (EX2_42 DEL)	-	-	AML+PKD	Yes (3)	Pathogenic	Novel	106.0
chr16:2098173-2138668 (EX2_42 DEL)	-	-	PKD	Yes (2)	Pathogenic	Novel	-
chr16:2112430-2136922 (EX13_38 DEL)	-	-	AML	No	Pathogenic	Novel	162.0
chr16:2120398-2121999 (EX17_19 DEL)	-	-	AML	No	Likely pathogenic	Novel	58.3
EX22_24 DEL (*)	-	-	AML	No	Likely pathogenic	Novel	112.9
c.5027_5068+32del	Splicing	p.Leu1676_Asp1690delinsHis	AML	No	Likely pathogenic	Novel	93.1

Notes: TSC, tuberous sclerosis complex; AML, angiomyolipoma; PKD, polycystic kidney disease. *The overlapped cases between the present study and the study of Cai et al. (2017). The number of affected family members was labeled.

The maximal diameters of AMLs in patients who underwent imaging evaluation in our hospital before any treatment were analyzed according to TSC gene mutations. All the probands and family members were included in the analysis. There was no statistically significant difference among AML maximal diameters between the *TSC1* mutation, *TSC2* mutation and NMI groups (58.5±29.0 vs. 107.3±60.6 vs. 86.9±53.5 mm, *P*= 0.309). When samples were grouped according to the pathogenicity of genetic mutations, there was no statistically significant difference between the pathogenic/likely pathogenic and benign/likely benign/NMI groups (105.5±59.5 vs. 90.4±58.4 mm, *P*= 0.363). When considering mutation type, no statistically significant difference was observed among the different *TSC2* mutation types of nonsense, missense, frameshift, splicing, and fragment deletion (*P*= 0.906) (Table 5). Moreover, no statistically significant difference in AML maximal diameter between the familial and sporadic groups was observed (105.1±66.3 vs. 100.5±56.7 mm, *P*= 0.775).

**Table 5 -:** Comparison of AML maximal diameters among different *TSC2* mutation types.

	Nonsense	Missense	Frameshift	Splicing	Fragment deletion	*P*
AML maximum diameter (mm)	101.7±51.1	113.5±69.1	118.6±78.0	94.1±39.0	101.5±35.7	0.906
range (mm)	31.0~204.0	30.2~211.0	14.0~266.0	58.0~146.4	58.3~162.0	-

Notes: TSC, tuberous sclerosis complex; AML, angiomyolipoma; PKD, polycystic kidney disease. *The overlapped cases between the present study and the study of Cai et al. (2017). The number of affected family members was labeled.

## Discussion

TSC is an autosomal dominant genetic disease that can also occur due to a sporadic germline mutation. The *TSC1* gene on chromosome 9q34 was first discovered in 1997 (van Slegtenhorst *et al.*, 1997), though the *TSC2* gene on chromosome 16p13.3 was discovered in 1993 (European Chromosome 16 Tuberous Sclerosis Consortium, 1993). The frequency of *TSC2* mutations is reported to be higher than that in *TSC1*, and when considering both familial and sporadic conditions, *TSC2* mutations are found in approximately 60% and *TSC1* mutations in approximately 19% of TSC patients (Kingswood et al., 2014). However, in 10~25% of TSC patients, *TSC1* or *TSC2* mutations cannot be detected by conventional genetic testing (Northrup *et al.*, 2013). Renal lesions in TSC patients mainly include AMLs and multiple renal cysts, whereas RCCs are relatively rare. Overall, AMLs are the most common renal features in TSC patients. Indeed, approximately 80% of TSC patients develop AMLs, which are significant causes of death. The risk of spontaneous bleeding of AML is related to the lesion volume, and approximately 25~50% of patients with AML diameters > 3~4 cm will experience hemorrhage (Aydin et al., 2009; Dixon et al., 2011). In addition to AMLs, renal cysts are relatively common TSC renal lesions. The *PKD1* gene is proximal to the *TSC2* gene on chromosome 16, and may lead to the possibility of TSC/PKD contiguous gene syndrome and the development of polycystic kidney disease (PKD) (Bissler and Kingswood, 2018). The patient in our study who harbored both *TSC2* and *PKD1* mutations presented a main phenotype of bilateral multiple renal cysts; his daughter had the same mutation and presented the same renal lesions. Nonetheless, another patient with *TSC2* and *PKD1* mutations showed both renal lesions of AMLs and PKD. The reasons for TSC patients developing multiple, bilateral RCCs remain unknown, and no other driver mutations have been identified in TSC-associated RCCs (Lam et al., 2018). The incidence of RCC in TSC patients is much lower than that of AML. It is approximately 4.4% in the Mayo Clinic cohort and 2.2% in the UK (Henske, 2004). There is one case with RCC in our study. RCCs in association with both *TSC1* and *TSC2* mutation have been reported (Carlo et al., 2019), though there are no exact data comparing *TSC1* and *TSC2* mutations. One patient with *TSC1* gene mutation in our study had bilateral Wilms tumors, the most common malignant renal tumor in children. Wilms tumor exhibits a high degree of genetic heterogeneity, and the related genes include *WT1* (chromosome 11p13), *WTX* (chromosome Xq11.1), *CTNMB1* (chromosome 3p22.1) and *TP53* (chromosome 17p13.1) (Scott et al., 2006). Spreafico *et al.* reported a girl with a *TSC2* mutation who developed a unilateral Wilms tumor. However, the girl was also found to carry mutations in the *WT1* and *WTX* genes (Spreafico *et al.*, 2011). However, the patient did not get a screening for the mutations of Wilms tumor. According to existing studies, it is likely that the occurrence of Wilms tumor is coincidental and that the conditions of TSC are not associated with an increased risk of Wilms tumor (Scott *et al.*, 2006).

*TSC2* mutations are usually related to more severe phenotypes than *TSC1* mutations (Peron et al., 2018a). The rate of *TSC1* mutations in our study was lower than the reported rate, and this may be because more patients with *TSC1* mutations had milder phenotypes and patients with *TSC2* mutations were more likely to seek treatment. According to previous studies, patients with *TSC2* mutations usually have large AML sizes and a high risk for AML hemorrhage (Cai et al., 2017; Li et al., 2018), whereas TSC patients with NMI are reported to have milder phenotypes than patients with *TSC2* mutations (Camposano et al., 2009). In our study, we compared AML maximal diameters between patients with *TSC1* mutation, *TSC2* mutation and NMI, and observed a trend of a higher average in those with *TSC2* mutations. Regardless, no statistically significant difference was found. However, in the study of Cai *et al.* (2017), the difference in AML maximal diameters between patients with *TSC2* mutations and non-TSC2 mutations was significant. In general, the different results may be due to the small sample sizes of patients in both studies. This study included most of the individuals in the 2017 report, and there were only 2 patients with NMI in the previous study. The maximal diameter in patients with NMI can be as large as 22.0 cm in our study, and the maximal diameter in patients with non-TSC2 mutations was 8.9 cm in Cai’s study, possibly affecting the statistical results.

In our study, 21.9% of probands of TSC patients were classified as NMI, and this rate is generally consistent with the literature (Peron et al., 2018b). In previous studies, mosaicism and intronic mutations have been detected by NGS in patients in whom no mutation was identified by conventional molecular diagnostic analysis of *TSC1* and *TSC2* (Tyburczy et al., 2015). Nonetheless, a significant proportion of patients with NMI in our study underwent NGS. However, no “*TSC* gene” other than *TSC1* or *TSC2* has been reported in the literature, and further research on the mechanisms is needed.

We also compared AML sizes among different kinds of mutation types. TSC gene mutations include nonsense mutations, missense mutations, small deletions or insertions, splice site changes and large deletions or rearrangements. Few studies have addressed such factors. Cai et al. (2017) reported AML sizes between patients with TSC2 mutations and non-TSC2 mutations. However, there were not enough details about mutation types. Here, we discuss the influence of different mutation types on the phenotypes of AML. Our results show no direct relationship between mutation type and renal phenotype severity. Nonsense mutations, small deletions or insertions, splice site changes and large deletions or rearrangements affect the integrity of the protein product. The human TSC2 protein contains 1807 residues and acts as a tumor suppressor in complex with TSC1. Three regions, the N terminal TSC1-interacting region (residues 55 to 469), tuberin type domain (residues 555 to 903) and GTPase activator domain (residues 1562 to 1748), are distinct according to sequence similarity searches with protein domain families (Sudarshan et al., 2019). Missense mutations in these regions will affect the function of the protein. We found that a change in tuberin function can also cause the same severe consequences as a change in tuberin integrity. However, further studies, including about protein structure and function, should be conducted in the future.

Typically, *TSC1* mutations are more likely to be familial than *TSC2* mutations (McEneaney and Tee, 2019; Jiangyi et al., 2020). This phenotypic diversity can be partly explained by the poorer prognosis of patients carrying *TSC2* mutations (Jiangyi *et al.*, 2020). In our study, two of four probands with *TSC1* mutation had a familial history, while eleven of fifty-three probands with *TSC2* were familial. Interestingly, two of 16 probands with NMI also presented familial disease. This indicates that inherited changes in genes may participate in disease onset, and further studies are needed to determine them.

The results may also be limited by the number of patients, which was too small to obtain a reliable statistical result in genotype-phenotype correlation. The frequency of *TSC1* mutation was 5.6% (4/73) in the probands, which is much lower than that reported in previous studies (Kingswood et al., 2014). This may be because fewer patients with *TSC1* mutations seek help due to only mild clinical manifestations. However, this result is consistent with Jiangyi’s study, which reported that Chinese TSC patients carry more TSC2 alterations than found in the TOSCA project (Jiangyi *et al.*, 2020). In general, studies with larger samples are needed to obtain more reliable results in the future.

## Conclusion

The relationship between the conditions of TSC genetic mutations and the type and severity of renal lesions still needs more study. Other focuses, such as protein structure and function, need to be addressed with regard to renal manifestations. Although *TSC1* and *TSC2* genetic mutations have been documented, patients with NMI, particularly those with familial disease, need more attention because the pathogenesis is unknown.
